# Clinical and pathophysiologic determinants of catheter ablation outcome in hypertrophic cardiomyopathy with atrial fibrillation

**DOI:** 10.1002/joa3.13061

**Published:** 2024-05-14

**Authors:** Jae‐Hyuk Lee, Iksung Cho, Sung Hwa Choi, Hee Tae Yu, Tae‐Hoon Kim, Jae‐Sun Uhm, Boyoung Joung, Moon‐Hyoung Lee, Geu‐Ru Hong, Chun Hwang, Hui‐Nam Pak

**Affiliations:** ^1^ Department of Cardiology, Myongji Hospital Hanyang University Medical Center Seoul Republic of Korea; ^2^ Yonsei University Health System Seoul Republic of Korea

**Keywords:** atrial fibrillation, atrial wall thickness, catheter ablation, hypertrophic cardiomyopathy

## Abstract

**Background:**

Hypertrophic cardiomyopathy (HCM) is frequently associated with atrial fibrillation (AF). We compared clinical, echocardiographic, and electrophysiological parameters between HCM subtypes and those without HCM at AF catheter ablation (AFCA) and analyzed post‐AFCA reverse remodeling and AF recurrence based on HCM presence and subtype.

**Methods:**

Among 5161 consecutive patients who underwent de novo AFCA, we included HCM patients and control patients who were age‐, gender‐, and AF type‐matched. Between AF‐HCM patients and controls, we compared baseline values for left atrium (LA) wall thickness (LAWT), reverse remodeling at 1‐year follow‐up, and procedural outcomes over the course of follow‐up between two groups.

**Results:**

A total of 122 AF‐HCM patients and 318 control patients were included. AF‐HCM patients had more frequent heart failure and higher LA diameter, E/Em, and LA pressure (all, *p* < .001). However, LAWT did not differ from control group. A year after AFCA, degree of LA reverse remodeling was significantly lower in AF‐HCM than in control group (ΔLA dimension, *p* = .025). Nonapical HCM (HR 1.71; 95% CI 1.05–2.80), persistent AF (HR 1.46; 95% CI 1.05–2.04), and LA dimension (HR 1.04; 95% CI 1.01–1.06) were independent risk factors for AF recurrence. During 78.0 months of follow‐up, nonapical HCM patients showed higher AF recurrence rate than both apical HCM (log‐rank *p* = .005) and control patients (log‐rank *p* = .002).

**Conclusions:**

The presence of HCM, particularly nonapical HCM, displayed increased LA hemodynamic loading with diastolic dysfunction and had poorer rhythm outcomes after AFCA compared to both apical HCM and control group.

## Graphical abstract

Comparison of LA hemodynamic and structural parameters between AF‐HCM and control groups and the difference of LA reservoir strain according to the type of HCM with rhythm outcome after AFCA in three groups.

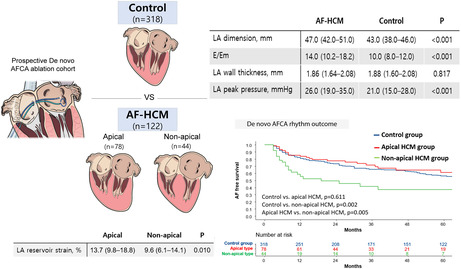

## 1 INTRODUCTION

Hypertrophic cardiomyopathy (HCM) is a relatively common genetic cardiac disease affecting one out of every 500 individuals in the general population and accompanies sarcomeric protein mutation in about 60% of the patients.^1^ Atrial fibrillation (AF) is the most frequent arrhythmia in HCM patients with the prevalence of 4–6 times higher than in an age‐matched general population.^2^ AF increases the risk of ischemic strokes by eightfold in HCM (AF‐HCM).^2^, ^3^, ^4^ Despite the recognition of AF catheter ablation (AFCA) as the most effective rhythm control strategy for patients with AF, most large‐scale studies evaluating its outcome have excluded those with AF‐HCM. This may be due to the complex interplay between AF‐HCM and the left ventricular (LV) hypertrophy, resulting in LV outflow tract (LVOT) obstruction and diastolic dysfunction, which have direct implications on the left atrial (LA) hemodynamic afterload and the development of remodeling myopathy.^5^, ^6^ In addition, potentially hypertrophied atrial wall, myofibril disarray, or microcirculatory ischemia‐associated atrial myopathy may contribute to the mechanism of AF‐HCM.^2^, ^7^, ^8^, ^9^, ^10^

The outcomes of AFCA in AF‐HCM patients have been evaluated in only a few clinical studies with inconsistent results, ranging from lower success rates and higher need for repeated procedures to equivalent success rates to those without structural heart disease.^2^, ^8^, ^11^, ^12^, ^13^, ^14^, ^15^, ^16^ This disparity in findings highlights the need for further investigation into the factors that may impact the effectiveness of AFCA in AF‐HCM patients. Therefore, in this study, we compared clinical, echocardiographic, and electrophysiological parameters between patients with and without HCM and among HCM subtypes (apical versus nonapical) at the time of AFCA, while also analyzing reverse remodeling and the recurrence of AF after the AFCA based on HCM presence and subtype.

## 2 METHODS

### 2.1 Study population

**FIGURE 1:**
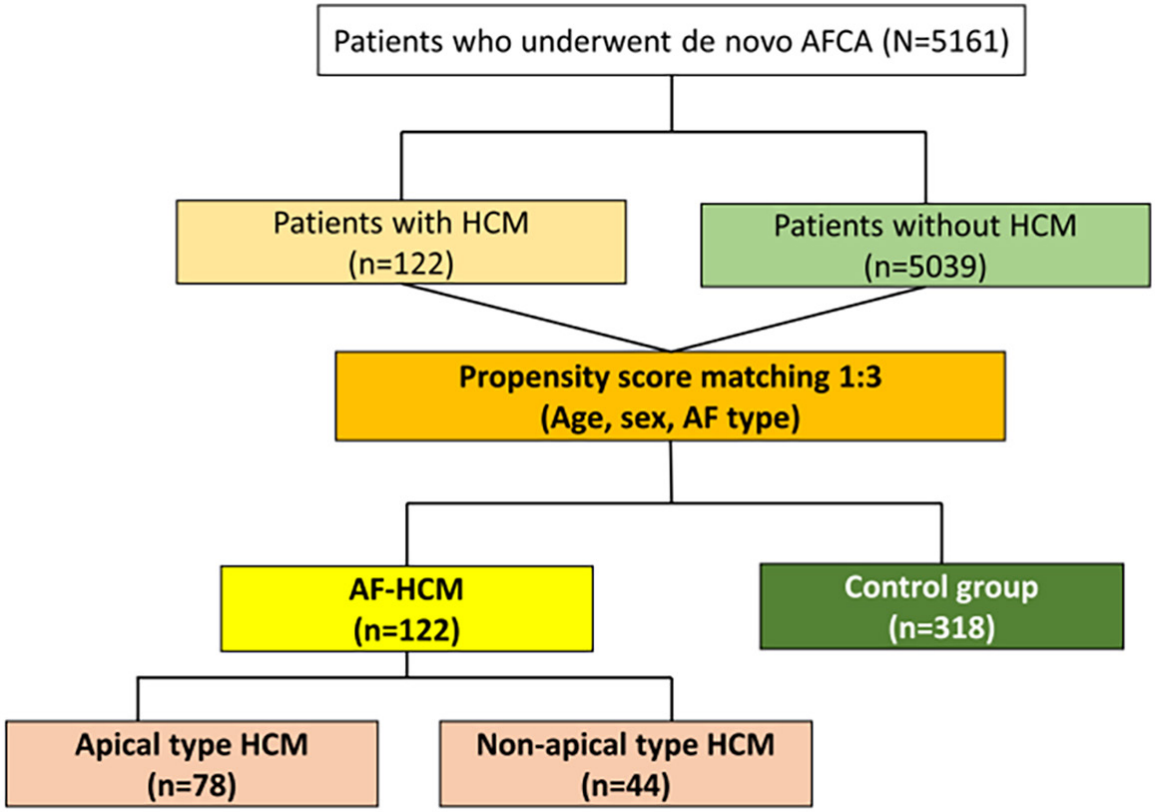
Patient selection and included patients of the study. AF, atrial fibrillation; AFCA, atrial fibrillation catheter ablation; HCM, hypertrophic cardiomyopathy.

The study protocol adhered to the principles of the Declaration of Helsinki and was approved by the Institutional Review Board of the Yonsei University Health System. All patients provided written informed consent for inclusion in the Yonsei AF Ablation Cohort (NCT02138695). Among 5161 prospectively enrolled consecutive patients who underwent de novo AFCA between March 2009 and September 2022 in a single center, we enrolled 122 patients with HCM (AF‐HCM group) and 318 propensity score‐matched patients (control group). Patients in the control group were selected from same cohort and were matched by age, gender, and type of AF (Figure 1). The exclusion criteria were as follows: (1) AF refractory to electrical cardioversion; (2) AF with rheumatic valvular disease; (3) a prior AF ablation or cardiac surgery; and (4) less than 18 years old. All patients stopped all anti‐arrhythmic drugs for a period corresponding to at least five half‐lives before the AFCA.

### 2.2 Echocardiography and definition of HCM

Transthoracic echocardiography (TTE) was conducted prior to the procedure (within 3 months) and at the 1‐year follow‐up. The HCM diagnosis was based on echocardiographic evidence of a hypertrophied and nondilated LV with a wall thickness ≥15 mm in the absence of another cardiac or systemic disease capable of producing a similar magnitude of hypertrophy.^17^ One‐year follow‐up TTE was conducted in 362 (80 in AF‐HCM group and 282 in control group) patients. We measured the echocardiographic parameters, including LA dimension, LA volume index, LV ejection fraction, and ratio of the peak mitral flow velocity of the early rapid filling to the early diastolic velocity of the mitral annulus (E/Em), and those parameters were compared between the baseline and 1‐year follow‐up TTE. In addition, HCM type according to the involvement location, presence of LVOT obstruction, and LV maximal thickness were evaluated in the AF‐HCM patients.

### 2.3 Two‐dimensional speckle‐tracking strain analysis

Speckle‐tracking strain analysis was performed using a dedicated semi‐automated software (AutoSTRAIN, TOMTEC‐ARENA, Munich, Germany) to assess LA function.^18^ LA strain was obtained from apical four‐ and two‐chamber images by semi‐automatic endocardial border tracking and manual adjustment to optimize tracking.^19^ Among the three phases of atrial strain (reservoir, conduit, and contractile), we investigate LA reservoir strain (LASr) at baseline and 1‐year follow‐up echocardiography to evaluate LA diastolic function in AF‐HCM patients.

### 2.4 Measurements of the LA wall thickness and LA pressure

Using pre‐AFCA cardiac computed tomography (CT), LA wall thickness (LAWT) was assessed by specialized software (AMBER, Laonmed Inc., Seoul, Korea).^20^, ^21^ The methodology and principles of this software have been previously described and have been validated through testing with a 3D printed phantom and in 120 patients.^20^, ^21^ In brief, the endocardium of the LA was semi‐automatically divided using the edge detector on the cardiac CT. The LA wall was then extracted with an overlapped area using morphology operations after separation from other tissues using the multi‐Otsu threshold algorithm in the Hounsfield unit histogram. After solving the vector field with Laplace's equation, the LAWT was calculated as a numerical streamline connecting the endocardium and epicardium using the Euler method. The thickness of the LA wall was measured at each segmented region (anterior wall, posterior wall, posterior‐inferior wall, interatrial septum, left lateral isthmus, and LA appendage) as well as in the overall LA chamber.

During the AFCA procedure, LA pressure was measured during sinus rhythm and AF immediately after trans‐septal puncture as described in previous studies. If the initial rhythm was AF, we terminated the AF rhythm by internal cardioversion, allowed the atrium at least 3 min to recover from the cardioversion's atrial stunning, and then assessed LA pressure during the ensuing sinus rhythm.^22^, ^23^ We excluded those patients in whom LA pressure during sinus rhythm could not be measured due to frequent re‐initiations of AF after electrical cardioversion.

### 2.5 Electrophysiological studies and catheter ablation

The electrophysiological mapping method and AFCA technique/strategy used during the study period were consistently performed as described previously.^24^ In brief, radiofrequency energy was delivered for the ablation under 3D electroanatomical mapping (NavX, Abbott, Inc., Minnetonka, MN, USA; CARTO system, Biosense Webster, Diamond Bar, CA, USA) using an open irrigated‐tip catheter (Flexibility, Abbott, USA; Smart‐touch, Celsius, ThermoCool, ThermoCool SF, Biosense Webster Inc., Diamond Bar, CA, USA). During high right atrial pacing at 500 ms, high‐quality voltage maps were obtained utilizing a circumferential mapping catheter. We collected 500–1000 direct contact bipolar electrograms from the LA endocardium and used peak–to–peak amplitude analysis to determine the mean LA electrogram voltage. An initial circumferential pulmonary vein (PV) isolation was performed on each patient. The roof line, posterior‐inferior line, anterior line, cavotricuspid isthmus line, superior vena cava to the septal line, or complex fractionated atrial electrogram‐guided ablation were added at the discretion of the operator. After cardioversion with isoproterenol infusion (5–20 μg/min; target heart rate, 120 bpm), the operation was deemed successful if there was no immediate return of AF. Extra‐PV foci for mappable AF triggers were mapped and abated to the greatest extent.

### 2.6 Postablation management and follow‐up

All patients visited the outpatient clinic at 1, 3, 6, and 12 months after the AFCA and every 6 months thereafter or whenever symptoms occurred. All patients underwent electrocardiography at every visit and 24‐h Holter recording at 3 and 6 months, then every 6 months for 2 years, annually at 2–5 years, and then biannually after 5 years according to the modified 2012 HRS/EHRA/ECAS expert consensus statement guidelines.^25^ AF recurrence was defined as any episode of AF or atrial tachycardia lasting at least 30 s. Any electrocardiographic documentation of AF recurrence 3 months after the blanking period was identified as clinical recurrence.

### 2.7 Statistical analysis

We used a propensity score approach to control confounding factors that might influence both group assignment and outcome. Age, gender, and type of AF were considered in the propensity score matching. We used a 1:3 matching algorithm, and the primary analysis was performed with selected subjects. Continuous variables are expressed as the mean ± standard deviation for normally distributed variables and as the median (interquartile range) for nonnormally distributed variables and were compared using Student's *t*‐test and Wilcoxon rank‐sum test, respectively. We used the chi‐square or Fisher's exact test to compare categorical variables. We conducted a Kaplan–Meier analysis test to analyze the probability of the freedom from AF recurrence after AFCA. Using Cox regression analysis, we identified predictors of AF recurrence after AFCA. The variables selected for the multivariate analysis were those with *p*‐value <.05 in the univariate analysis. We used Statistical Package for the Social Sciences version 25.0 (IBM Corporation, Armonk, NY, USA) and R software version 3.6.2 (The R Foundation for Statistical Computing, Vienna, Austria) for data analysis.

## 3 RESULTS

### 3.1 Clinical and imaging characteristics of AF‐HCM

Among 5161 consecutive patients (73.9% male, 60.0 [52.0–67.0] years, 63.8% paroxysmal AF) who underwent de novo AFCA, we enrolled 122 patients with HCM and 318 in the matched control group (78.6% male, 61.0 [56.0–67.0] years, 57.3% paroxysmal AF, Table 1). In AF‐HCM group, 78 patients (63.9%) had apical HCM, and 44 patients (36.1%) had nonapical HCM. After propensity score matching, the AF‐HCM group had a higher proportion of heart failure (*p* < .001), and higher LA dimension (*p* < .001), and E/Em (*p* < .001) than the control group (Table 1). However, mean LAWT (*p* = .817) and each regional LAWT did not differ between the two groups (Table 1; Table S1).

**TABLE 1:** Baseline clinical characteristics between AF‐HCM and control groups.

	All subjects (*n* = 440)	AF‐HCM (*n* = 122)	Control (*n* = 318)	*p*
Age (year)	61.0 (56.0–67.0)	61.0 (56.0–67.0)	61.0 (56.0–66.0)	.758
Male gender, *n* (%)	346 (78.6)	98 (80.3)	248 (78.0)	.685
Paroxysmal AF, *n* (%)	252 (57.3)	68 (55.7)	184 (57.9)	.768
AF duration (month)	24.5 (10.0–48.0)	22.0 (8.0–48.0)	27.5 (12.0–50.0)	.128
Body mass index (kg/m^2^)	24.6 (23.0–26.6)	24.8 (23.3–26.8)	24.5 (22.9–26.6)	.281
Comorbidity, *n* (%)				
Hypertension	242 (55.0)	66 (54.1)	176 (55.3)	.898
Diabetes	77 (17.5)	15 (12.3)	62 (19.5)	.101
Stroke/TIA	63 (14.3)	20 (16.4)	43 (13.5)	.537
Heart failure	75 (17.0)	42 (34.4)	33 (10.4)	<.001
Vascular disease	79 (18.0)	17 (13.9)	62 (19.5)	.222
CHA_2_DS_2_VASc	2.0 (1.0–3.0)	3.0 (2.0–4.0)	2.0 (1.0–3.0)	<.001
Echocardiographic findings				
LA dimension (mm)	44.0 (40.0–48.0)	47.0 (42.0–51.0)	43.0 (38.0–46.0)	<.001
LV ejection fraction (%)	64.0 (59.0–70.0)	67.0 (62.0–72.0)	63.0 (59.0–69.0)	<.001
E/Em	10.8 (8.0–14.0)	14.0 (10.2–18.2)	10.0 (8.0–12.0)	<.001
Mean LA wall thickness (mm)	1.86 (1.64–2.08)	1.86 (1.64–2.08)	1.88 (1.60–2.08)	.817

*Note*: Values are presented as median (Q1–Q3 quartiles [25th and 75th percentiles]) or number (%).

Abbreviations: AF, atrial fibrillation; AF‐HCM, atrial fibrillation with hypertrophic cardiomyopathy; E/Em, ratio of the peak mitral flow velocity of the early rapid filling to the early diastolic velocity of the mitral annulus; LA, left atrium; LV, left ventricular; TIA, transient ischemic attack.

### 3.2 Intraprocedural and procedural findings of AF‐HCM

During the procedure, we investigated intraprocedural values in two groups. AF‐HCM group had higher LA peak pressure (*p* < .001) and mean LA voltage (*p* = .011) than control group, but the existence of extra‐PV foci did not differ between two groups (*p* = .475, Table 2). Empirical extra‐PV LA ablation was more conducted in control group than AF‐HCM group (*p* < .001, Table 2). Furthermore, procedure‐related complication rate (*p* = .981) and incident stroke rate during follow‐up periods (*p* = .876) did not differ between the AF‐HCM and control groups (Table 3).

**TABLE 2:** Intraprocedural findings in the AF‐HCM and control groups.

	All subjects (*n* = 440)	AF‐HCM (*n* = 122)	Control (*n* = 318)	*p*
Intraprocedural findings				
LA peak pressure (mmHg)	23.0 (17.0–31.0)	26.0 (19.0–35.0)	21.0 (15.0–28.0)	<.001
Mean LA voltage (mV)	1.2 (0.7–1.6)	1.4 (0.8–1.9)	1.1 (0.7–1.6)	.011
Extra‐PV foci, *n* (%)	24 (7.3)	8 (9.6)	16 (6.5)	.475
Ablation lesion, *n* (%)				
CPVI	440 (100)	122 (100)	318 (100)	1
Empirical extra‐PV LA ablation	213 (48.4)	42 (34.4)	171 (53.8)	<.001
Posterior box isolation	161 (36.6)	32 (26.2)	129 (40.6)	.007
Left lateral isthmus	24 (5.5)	4 (3.3)	20 (6.3)	.312
Anterior line	147 (33.4)	25 (20.5)	122 (38.4)	.001
CFAE	29 (6.6)	3 (2.5)	26 (8.2)	.051

*Note*: Values are presented as median (Q1–Q3 quartiles [25th and 75th percentiles]) or number (%).

Abbreviations: AF‐HCM, atrial fibrillation with hypertrophic cardiomyopathy; CFAE, complex fractionated atrial electrograms; CPVI, circumferential pulmonary vein isolation; LA, left atrium; PV, pulmonary vein.

**TABLE 3:** Procedural outcomes during follow‐up and 1‐year follow‐up and changes of echocardiographic findings in the AF‐HCM and control groups.

	All subjects (*n* = 440)	AF‐HCM (*n* = 122)	Control (*n* = 318)	*p*
Procedural outcomes, *n* (%)				
Post‐AFCA anti‐arrhythmic agent use	204 (46.4)	60 (49.2)	144 (45.3)	.280
Early recurrence	125 (28.4)	31 (25.4)	94 (29.6)	.456
Clinical recurrence	205 (46.6)	49 (40.2)	156 (49.1)	.117
Recurrence as AF	141 (68.8)	32 (65.3)	109 (69.9)	
Recurrence as AT	64 (31.2)	17 (34.7)	47 (30.1)	
Complication	20 (4.5)	5 (4.1)	15 (4.7)	.981
Incident stroke during follow‐up	9 (2.1)	3 (2.8)	6 (1.9)	.876

	Overall (*n* = 362)	AF‐HCM (*n* = 80)	Control (*n* = 282)	*p*
1‐year f/u echocardiography				
LA dimension (mm)	40.0 (36.0–44.0)	44.0 (40.0–48.0)	39.0 (36.0–43.0)	<.001
LV ejection fraction (%)	65.0 (61.0–70.0)	66.0 (62.0–69.5)	64.0 (60.0–70.0)	.083
E/Em	10.0 (8.0–14.8)	14.5 (11.0–20.7)	10.0 (8.0–13.0)	<.001
1‐year change in the parameters compared to baseline echocardiography				
ΔLA dimension (mm)	−3.0 (−6.0–0)	−2.0 (−4.5–0)	−3.0 (−6.0–0)	.025
ΔLV ejection fraction (%)	1.0 (−3.0–6.0)	0 (−4.5–4.0)	1.0 (−3.0–6.0)	.201
ΔE/Em	0.1 (−1.7–2.7)	1.0 (−1.1–4.0)	0 (−1.9–2.0)	.035

*Note*: Values are presented as median (Q1–Q3 quartiles [25th and 75th percentiles]) or number (%).

Abbreviations: AF‐HCM, atrial fibrillation with hypertrophic cardiomyopathy; AFCA, atrial fibrillation catheter ablation; AT, atrial tachycardia; E/Em, ratio of the peak mitral flow velocity of the early rapid filling to the early diastolic velocity of the mitral annulus; LA, left atrium; LV, left ventricular.

### 3.3 Atrial reverse remodeling in HCM

**FIGURE 2:**
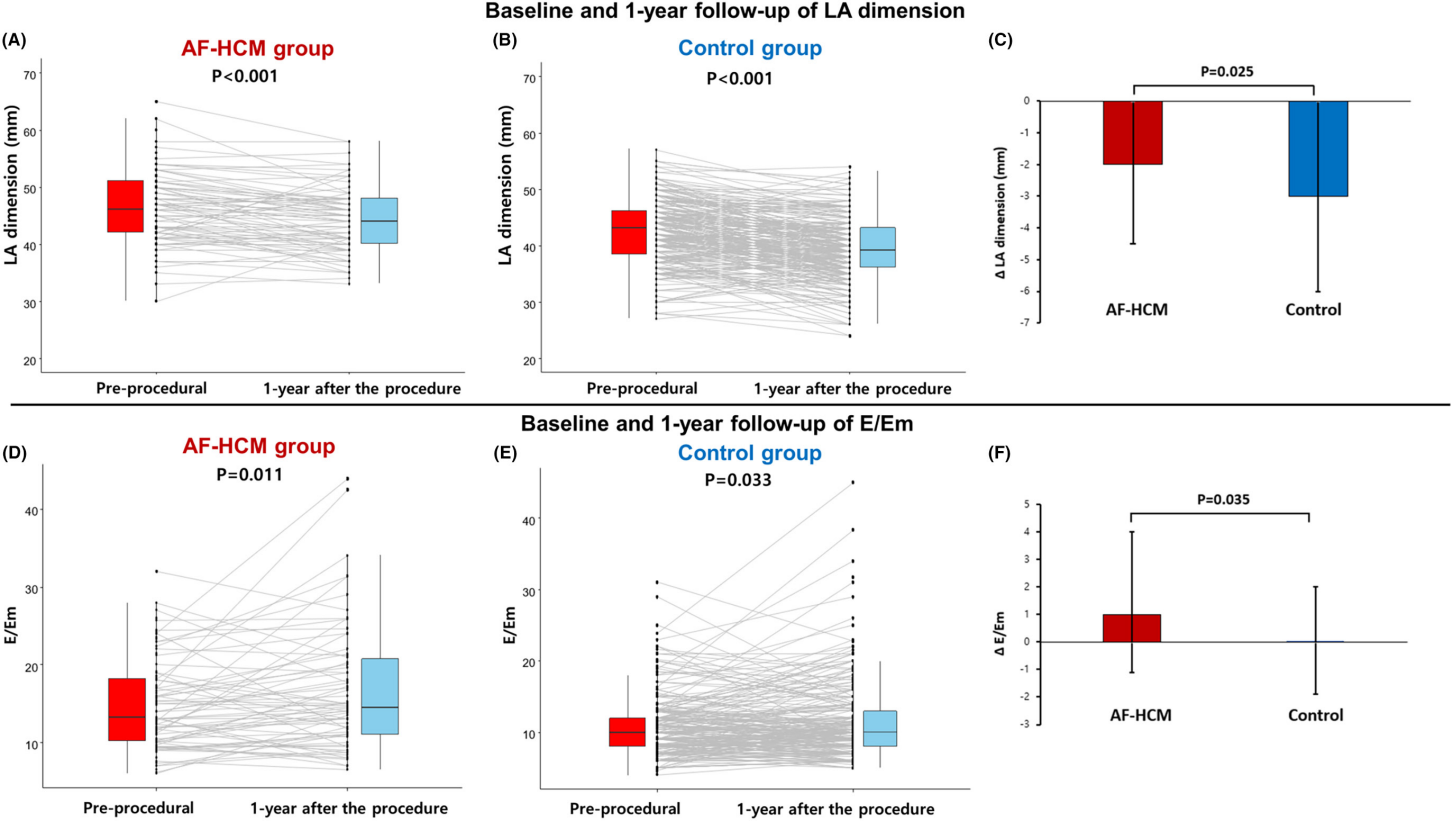
Changes in the echocardiography parameters before and 1 year after the AFCA depending on the presence of HCM. Although LA dimension was significantly reduced 1‐year after the AFCA in both the AF‐HCM group (A) and control group (B), the change in the LA dimension was significantly lower in the AF‐HCM group than control group (C). E/Em was increased in both the AF‐HCM group (D) and control group (E), but the degree of the E/Em increase was significantly higher in the AF‐HCM (F). AF, atrial fibrillation; AFCA, atrial fibrillation catheter ablation; E/Em, ratio of the peak mitral flow velocity of the early rapid filling to the early diastolic velocity of the mitral annulus; HCM, hypertrophic cardiomyopathy; LA, left atrium.

We compared echocardiographic parameters between baseline and 1‐year after the procedure between AF‐HCM and control groups. Although AFCA reduced LA dimension significantly a year after the procedure in both AF‐HCM and control groups (Figure 2A,B), the degree of the LA size reduction was significantly lower in the AF‐HCM group than control group (−2.0 [−4.5–0] vs. −3.0 [−6.0–0], *p* = .025; Table 3; Figure 2C). Similarly, E/Em was increased a year after the procedure in both groups (Figure 2D,E), but the degree of the E/Em increase was significantly higher in the AF‐HCM group than control group (1.0 [−1.1–4.0] vs. 0 [−1.9–2.0], *p* = .035; Table 3; Figure 2F). We further assessed LA reverse remodeling between two groups based on the recurrence of AF. In subjects without AF recurrence, the extent of the LA size reduction was significantly lower in the AF‐HCM group compared to the control group. However, in subjects with AF recurrence, there was no significant difference in LA reverse remodeling between the two groups (Tables S2 and S3).

### 3.4 AFCA rhythm outcome and associated factors with AF recurrence

**FIGURE 3:**
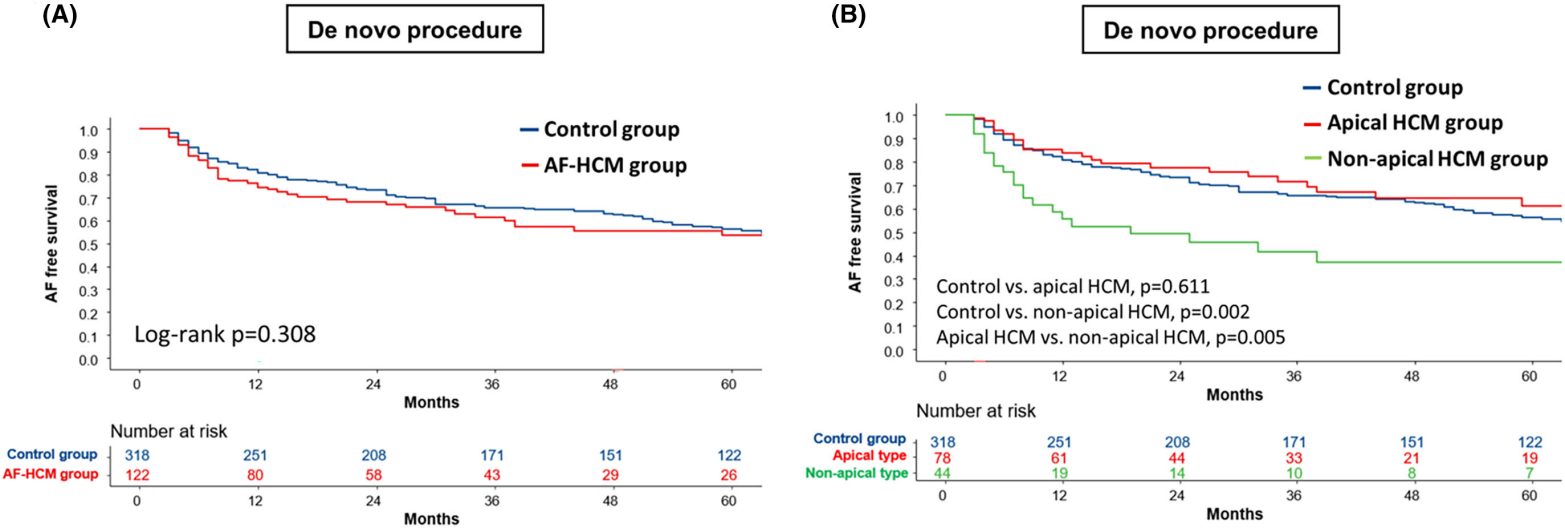
Kaplan–Meier curve for the clinical recurrence of AF after de novo and repeat procedure. (A) AF recurrence rate did not differ between AF‐HCM and control groups (log‐rank *p* = .308). (B) AF recurrence rate was significantly higher in the nonapical type HCM group than both apical type HCM group (log‐rank *p* = .005) and control group (log‐rank *p* = .002). AF, atrial fibrillation; AFCA, atrial fibrillation catheter ablation; HCM, hypertrophic cardiomyopathy.

During a median follow‐up of 78.0 (IQR 36.3–120.8) months, clinical recurrence was observed in 46.6% of patients. The rate of clinical recurrence between the AF‐HCM and control groups was comparable (log‐rank *p* = .308; Figure 3A). Yet, when HCM was divided into apical and nonapical types, the nonapical HCM group had a significantly higher recurrence rate than both control (log‐rank *p* = .002) and apical HCM groups (log‐rank *p* = .005) (Figure 3B). Importantly, there was no significant difference in the AF recurrence rate between the apical HCM and the control group (log‐rank *p* = .611).

In the multivariate Cox regression analysis to identify risk factors for AF recurrence, nonapical HCM was an independent risk factor (HR 1.71; 95% CI 1.05–2.80; *p* = .031), after adjusting other clinical and electrophysiological risk factors for the recurrence such as persistent AF, LA dimension, and empirical extra‐PV LA ablation (Table 4). Additionally, persistent AF (HR 1.46; 95% CI 1.05–2.04; *p* = .025) and LA dimension (HR 1.04; 95% CI 1.01–1.06; *p* = .006) were also identified as independent risk factors for AF recurrence. In contrast, apical HCM did not stand out as an independent predictor for AF recurrence (HR 0.83; 95% CI 0.55–1.25; *p* = .375).

**TABLE 4:** Cox regression analysis for AF recurrence in included subjects.

	Multivariate	
	Adjusted HR (95% CI)^a^	*p*
Nonapical HCM	1.71 (1.05–2.80)	.031
Persistent AF	1.46 (1.05–2.04)	.025
LA dimension	1.04 (1.01–1.06)	.006

Abbreviations: AF, atrial fibrillation; CI, confidence interval; HCM, hypertrophic cardiomyopathy; HR, hazard ratio; LA, left atrium.

^a^ HRs were adjusted for persistent AF, LA dimension, empirical extra‐PV LA ablation, and nonapical type HCM those were with *p*‐value <.05 in the univariate analysis.

### 3.5 Characteristics and outcome according to the type of HCM

Since nonapical type HCM was an independent factor for AF recurrence, we investigated clinical factors and procedural outcomes in the AF‐HCM subset according to the type of HCM. The nonapical HCM group had a higher proportion of heart failure (*p* = .012), LVOT obstruction (<.001), a higher LA dimension (*p* = .028), E/Em (*p* = .001), LV max thickness (*p* = .001), and LA peak pressure (*p* = .003) than apical HCM (Table 5). Importantly, LASr was significantly lower in patients with nonapical HCM compared to those with apical HCM (9.6% [6.1–14.1] vs. 13.7% [9.8–18.8], *p* = .010, Table 5).

**TABLE 5:** Clinical and procedural characteristics in subgroups according to the type of HCM in AF‐HCM group.

	Apical HCM (*n* = 78)	Nonapical HCM (*n* = 44)	*p*
Age (year)	62.0 (56.0–68.0)	60.5 (55.0–65.5)	.200
Male gender, *n* (%)	65 (83.3)	33 (75.0)	.382
Paroxysmal AF, *n* (%)	45 (57.7)	23 (52.3)	.697
AF duration (month)	24.0 (8.0–60.0)	20.0 (7.0–36.0)	.344
Body mass index (kg/m^2^)	25.0 (23.9–27.0)	24.6 (22.5–25.7)	.095
Comorbidity, *n* (%)			
Hypertension	44 (56.4)	22 (50.0)	.622
Diabetes	11 (14.1)	4 (9.1)	.601
Stroke/TIA	11 (14.1)	9 (20.5)	.512
Heart failure	20 (25.6)	22 (50.0)	.012
Vascular disease	12 (15.4)	5 (11.4)	.731
CHA_2_DS_2_VASc	3.0 (2.0–4.0)	3.0 (2.0–3.5)	.735
Echocardiographic findings			
LA dimension (mm)	45.0 (41.0–50.0)	48.0 (44.0–52.5)	.028
LV ejection fraction (%)	67.5 (63.0–71.0)	66.0 (58.5–73.5)	.257
E/Em	12.7 (9.4–17.0)	16.5 (12.5–21.5)	.001
LVOT or mid‐ventricular obstruction	1^a^ (1.3)	15 (34.1)	<.001
LV max thickness	17.0 (15.0–19.0)	20.0 (16.5–21.5)	.001
LA reservoir stain (%)	13.7 (9.8–18.8)	9.6 (6.1–14.1)	.010
Mean LA wall thickness	1.89 (1.74–2.08)	1.81 (1.53–2.02)	.241
Intraprocedural findings			
LA peak pressure (mmHg)	25.0 (17.0–32.5)	34.0 (24.0–38.0)	.003
Mean LA voltage (mV)	1.4 (0.9–1.9)	1.4 (0.7–2.1)	.754
Extra‐PV foci	8 (13.1)	0 (0)	.172

*Note*: Values are presented as median (Q1–Q3 quartiles [25th and 75th percentiles]) or number (%).

Abbreviations: AF, atrial fibrillation; AF‐HCM, atrial fibrillation with hypertrophic cardiomyopathy; CFAE, complex fractionated atrial electrograms; CPVI, circumferential pulmonary vein isolation; E/Em, ratio of the peak mitral flow velocity of the early rapid filling to the early diastolic velocity of the mitral annulus; LA, left atrium; LV, left ventricular; PV, pulmonary vein; TIA, transient ischemic attack.

^a^ Mid‐ventricular obstruction.

In the multivariate Cox regression analysis to identify risk factors for AF recurrence in HCM group, nonapical HCM (HR 2.28; 95% CI 1.26–4.16; *p* = .007) and persistent AF (HR 2.02; 95% CI 1.09–3.74; *p* = .025) were independent risk factors, after adjusting other clinical and electrophysiological risk factors for the recurrence (Table S4).

We further examined the recurrence rate based on the type of AF within the HCM population. Within the AF‐HCM group, clinical recurrence rate was significantly higher in those with persistent AF than paroxysmal AF (log‐rank *p* = .016; Figure S1). For those with persistent AF, the recurrence rate across the three groups—apical HCM, nonapical HCM, and the control group—was consistent with the statistically significant results of the overall study groups. However, for paroxysmal AF, while the recurrence rate trended similarly, it did not achieve statistical significance (Figure S2A,B).

### 3.6 Repeat procedural findings in two groups

Among 205 patients who experienced AF recurrences, 28 patients underwent a repeat AFCA (10 in the AF‐HCM group [8 apical and 2 nonapical type] and 18 in the control group, Table S5). Time interval between de novo and repeat procedures (*p* = .517), PV reconnection rate (*p* = .755), and the existence of extra‐PV triggers (*p* = .546) did not differ between the two groups (Table S5). There was no significant difference in AF recurrence rate between the two groups during a 19.0 (12.0–27.5) month follow‐up period (log‐rank *p* = .495; Figure S3).

## 4 DISCUSSION

### 4.1 Main findings

In this prospective registry data on AFCA, we sought to explore the differences in clinical, echocardiographic, and electrophysiological parameters between patients with HCM and those without, and among HCMP subtypes. We also examined the patterns of clinical AF recurrence and post‐AFCA atrial reverse remodeling based on the presence and subtype of HCM. Compared to those without HCM, AF‐HCM patients exhibited a larger LA size and elevated LA pressure at the time of AFCA. AF‐HCM patients demonstrated less favorable LA reverse remodeling after AFCA over a 1‐year follow‐up. While clinical AF recurrence did not differ based on the presence of HCM during the follow‐up, patients with nonapical HCM experienced a significantly higher recurrence rate compared to the control group, which in contrast was not observed in those with apical HCM.

### 4.2 Rhythm control for AF‐HCM

Rhythm control in AF‐HCM patients is a valuable option, but there is controversy whether the effect of AFCA is inferior to that in non‐HCM patients. Some studies have reported lower success rates and higher requirements for repeat AFCA procedures in AF‐HCM patients, and it might be attributable to a hypertrophied LA wall.^2^, ^8^, ^11^, ^12^, ^13^, ^14^, ^16^ Contrary to the previous reports, we observed similar rhythm outcomes after the AFCA in patients with HCM as compared to those without. In addition, our study showed that the presence of HCM did not affect wall thickness of LA, which was consistent with a previous study by Hayashi et al.^15^ Furthermore, our study examined the impact of the type of HCM on the rhythm outcome, and surprisingly, the recurrence rate between the apical HCM group and the control group did not differ significantly, while we observed that nonapical HCM patients had a worse rhythm outcome than both the apical HCM and control groups. The relatively high proportion of apical HCM patients in our cohort may have contributed to the similar rhythm outcome between the HCM and non‐HCM groups. Although we adjusted for some potential confounding factors during comparison between the AF‐HCM group and the control group, we still observed that nonapical HCM type is independent factors for AF recurrence. These findings suggest that the type of HCM may play an important role in predicting the rhythm outcome after AFCA.

### 4.3 Apical vs. nonapical HCM

Apical HCM is more prevalent in Asian countries than in Western countries and is associated with a lower myocardial fibrosis burden, less diastolic dysfunction, and a benign clinical course compared to nonapical HCM.^26^, ^27^ In this study, two‐thirds of the patients had apical type HCM and only 13.1% of AF‐HCM patients had LVOT or mid‐ventricular obstruction, which has a relatively good prognosis. Therefore, the selection bias toward apical HCM patients in our study may have influenced the rhythm outcome of AFCA in AF‐HCM patients.

In this study, we measured the LASr in the HCM group to explore the potential mechanisms underlying the difference in AF recurrence between patients with apical‐ and nonapical HCM. LASr is a measure of the LA function that assesses the deformation of the LA wall during ventricular systole.^28^ Our results revealed that nonapical HCM patients had lower LASr values than apical HCM patients. Apical HCM is characterized by hypertrophy of the apex of the heart, which can lead to less myocardial fibrosis, less diastolic dysfunction, and less increased LV filling pressure compared to nonapical HCM. The difference in LASr between apical and nonapical HCM may be due to the structural and functional differences between the two types of HCM.^26^ Other possible factors that may influence LASr in HCM patients include the distribution and severity of hypertrophy, the presence of genetic mutations, and comorbidities. However, more research is needed to fully understand the mechanisms underlying the differences in LASr between apical and nonapical HCM patients.

### 4.4 Repeat procedure in AF‐HCM

Previous studies reported that AFCA is still effective choice for maintaining sinus rhythm in HCM patients although AFCA is not as efficacious in HCM patients compared to AF patients with no structural heart disease.^14^, ^29^ In this study, we investigate repeat procedural findings in AF‐HCM and control groups, and the results revealed that the PV reconnection rate and the existence of extra‐PV triggers during the repeat procedure did not differ between two groups. These findings might suggest that LV diastolic function may have played a more significant role in the hemodynamics and reverse remodeling of the LA and may have affected AF recurrence rate after de novo AFCA in AF patients with HCM.

### 4.5 Limitations

This study had several limitations. First, this was an observational prospective cohort study of highly selected patients who underwent AFCA. Moreover, there could be selection bias because this study was not an all‐comers design ablation study. We tried to resolve some limitations by selecting propensity score‐matched control groups. Second, the differentiation of HCM from other infiltrative heart diseases, such as cardiac amyloidosis, was not performed in a prospective manner for all patients using methods such as CMR, genetic studies, or myocardial biopsy. Instead, these diagnostic tests were conducted at the discretion of the treating clinicians on an as‐needed basis. This approach may have resulted in some patients with infiltrative heart diseases being included in the HCM group, potentially affecting the study findings. Third, although we kept a consistent ablation protocol performed by experienced operators, the catheter and mapping technologies kept changing during the long enrollment period. Fourth, the evaluation of LA size by the LA diameter could have been limited than by the LA volume, but since all the patients underwent serial echocardiography before and 1 year after the procedure, we could evaluate the changes in LA size and compare the differences between two groups with LA diameter. Fifth, more frequently performed additional extra‐PV LA ablation in control group would be one of factor that influenced AF recurrence outcome. However, we found that the apical type HCM still remained as independent factor for AF recurrence even after adjusting ablation strategy during Cox regression analysis. Sixth, previous studies reported that the proportion of the low voltage area in LA is associated with extent of atrial fibrosis and AF recurrence. However, our study evaluated LA voltage value with a parameter of mean LA voltage and this parameter would have limited value to reflect atrial fibrosis. Seventh, we have no data of pre‐ and postprocedural AF burden. Eighth, although HCM is a genetic disease, we did not evaluate the genetic susceptibility for HCM, genetic influence on the HCM type, or outcome of AFCA. However, recent studies reported that there has been no significant impact on genetic mutation on ablation outcomes.^12^ Further study including a genetic study in HCM is necessary.

## 5 CONCLUSION

In this study, AF‐HCM patients showed increased LA hemodynamic loading and diastolic dysfunction, but similar post‐AFCA rhythm outcomes compared to the control group. Yet, nonapical HCM type is important poor prognostic factor and the nonapical HCM patients had less favorable rhythm outcome compared to both non‐HCM and apical HCM patients.

## CONFLICT OF INTEREST STATEMENT

The authors declare no conflict of interest.

## APPROVAL OF THE RESEARCH PROTOCOL

The study was approved by the institutional review board.

## INFORMED CONSENT

Informed consent was obtained from all patients at admission.

## REGISTRY AND THE REGISTRATION NUMBER

N/A.

## ANIMAL STUDIES

N/A.

## Supporting information


Appendix S1.

## References

[1] Maron BJ , Maron MS , Semsarian C . Genetics of hypertrophic cardiomyopathy after 20 years: clinical perspectives. J Am Coll Cardiol. 2012;60(8):705–715. 22796258 10.1016/j.jacc.2012.02.068

[2] Providencia R , Elliott P , Patel K , McCready J , Babu G , Srinivasan N , et al. Catheter ablation for atrial fibrillation in hypertrophic cardiomyopathy: a systematic review and meta‐analysis. Heart. 2016;102(19):1533–1543. 27234160 10.1136/heartjnl-2016-309406

[3] Olivotto I , Cecchi F , Casey SA , Dolara A , Traverse JH , Maron BJ . Impact of atrial fibrillation on the clinical course of hypertrophic cardiomyopathy. Circulation. 2001;104(21):2517–2524. 11714644 10.1161/hc4601.097997

[4] Guttmann OP , Pavlou M , O'Mahony C , Monserrat L , Anastasakis A , Rapezzi C , et al. Prediction of thrombo‐embolic risk in patients with hypertrophic cardiomyopathy (HCM risk‐CVA). Eur J Heart Fail. 2015;17(8):837–845. 26183688 10.1002/ejhf.316 PMC4737264

[5] Maron BJ , Haas TS , Maron MS , Lesser JR , Browning JA , Chan RH , et al. Left atrial remodeling in hypertrophic cardiomyopathy and susceptibility markers for atrial fibrillation identified by cardiovascular magnetic resonance. Am J Cardiol. 2014;113(8):1394–1400. 24589281 10.1016/j.amjcard.2013.12.045

[6] Yoshida K , Hasebe H , Tsumagari Y , Tsuneoka H , Ebine M , Uehara Y , et al. Comparison of pulmonary venous and left atrial remodeling in patients with atrial fibrillation with hypertrophic cardiomyopathy versus with hypertensive heart disease. Am J Cardiol. 2017;119(8):1262–1268. 28214001 10.1016/j.amjcard.2016.12.025

[7] Alasady M , Shipp NJ , Brooks AG , Lim HS , Lau DH , Barlow D , et al. Myocardial infarction and atrial fibrillation: importance of atrial ischemia. Circ Arrhythm Electrophysiol. 2013;6(4):738–745. 23873140 10.1161/CIRCEP.113.000163

[8] Di Donna P , Olivotto I , Delcre SD , Caponi D , Scaglione M , Nault I , et al. Efficacy of catheter ablation for atrial fibrillation in hypertrophic cardiomyopathy: impact of age, atrial remodelling, and disease progression. Europace. 2010;12(3):347–355. 20173211 10.1093/europace/euq013

[9] Guttmann OP , Pavlou M , O'Mahony C , Monserrat L , Anastasakis A , Rapezzi C , et al. Predictors of atrial fibrillation in hypertrophic cardiomyopathy. Heart. 2017;103(9):672–678. 27794017 10.1136/heartjnl-2016-309672

[10] Ohtani K , Yutani C , Nagata S , Koretsune Y , Hori M , Kamada T . High prevalence of atrial fibrosis in patients with dilated cardiomyopathy. J Am Coll Cardiol. 1995;25(5):1162–1169. 7897130 10.1016/0735-1097(94)00529-y

[11] Contreras‐Valdes FM , Buxton AE , Josephson ME , Anter E . Atrial fibrillation ablation in patients with hypertrophic cardiomyopathy: long‐term outcomes and clinical predictors. J Am Coll Cardiol. 2015;65(14):1485–1487. 25857916 10.1016/j.jacc.2014.12.063

[12] Creta A , Elliott P , Earley MJ , Dhinoja M , Finlay M , Sporton S , et al. Catheter ablation of atrial fibrillation in patients with hypertrophic cardiomyopathy: a European observational multicentre study. Europace. 2021;23(9):1409–1417. 33930121 10.1093/europace/euab022

[13] Ezzeddine FM , Agboola KM , Hassett LC , Killu AM , Munoz FD , DeSimone CV , et al. Catheter ablation of atrial fibrillation in patients with and without hypertrophic cardiomyopathy: systematic review and meta‐analysis. Europace. 2023;25:euad256. 37595138 10.1093/europace/euad256 PMC10498139

[14] Faraz F , Rehman MEU , Sabir B , Ghaffar A , Iftikhar A , Maqsood A , et al. Efficacy of catheter ablation for atrial fibrillation in hypertrophic cardiomyopathy: a systematic review and meta‐analysis. Curr Probl Cardiol. 2023;48(3):101524. 36455792 10.1016/j.cpcardiol.2022.101524

[15] Hayashi H , Hayashi M , Miyauchi Y , Takahashi K , Uetake S , Tsuboi I , et al. Left atrial wall thickness and outcomes of catheter ablation for atrial fibrillation in patients with hypertrophic cardiomyopathy. J Interv Card Electrophysiol. 2014;40(2):153–160. 24763706 10.1007/s10840-014-9894-y

[16] Latif A , Ahmad S , Ahsan MJ , Willman C , Lateef N , Kapoor V , et al. Catheter ablation of atrial fibrillation in hypertrophic cardiomyopathy: a proportional meta‐analysis and systematic review of single‐arm studies. Heart Rhythm O2. 2023;4(4):258–267. 37124551 10.1016/j.hroo.2023.01.002 PMC10134396

[17] Ommen SR , Mital S , Burke MA , Day SM , Deswal A , Elliott P , et al. 2020 AHA/ACC guideline for the diagnosis and treatment of patients with hypertrophic cardiomyopathy: a report of the American College of Cardiology/American Heart Association Joint Committee on Clinical Practice Guidelines. J Am Coll Cardiol. 2020;76(25):e159–e240. 33229116 10.1016/j.jacc.2020.08.045

[18] Romanowicz J , Ferraro AM , Harrington JK , Sleeper LA , Adar A , Levy PT , et al. Pediatric normal values and Z score equations for left and right ventricular strain by two‐dimensional speckle‐tracking echocardiography derived from a large cohort of healthy children. J Am Soc Echocardiogr. 2022;36:310–323. 36414123 10.1016/j.echo.2022.11.006

[19] Badano LP , Kolias TJ , Muraru D , Abraham TP , Aurigemma G , Edvardsen T , et al. Standardization of left atrial, right ventricular, and right atrial deformation imaging using two‐dimensional speckle tracking echocardiography: a consensus document of the EACVI/ASE/Industry Task Force to standardize deformation imaging. Eur Heart J Cardiovasc Imaging. 2018;19(6):591–600. 29596561 10.1093/ehjci/jey042

[20] Kwon O‐S , Lee J , Lim S , Park J‐W , Han H‐J , Yang S‐H , et al. Accuracy and clinical feasibility of 3D‐myocardial thickness map measured by cardiac computed tomogram. Int J Arrhythm. 2020;21(1):12.

[21] Lee JH , Yu HT , Kwon OS , Han HJ , Kim TH , Uhm JS , et al. Atrial wall thickness and risk of hemopericardium in elderly women after catheter ablation for atrial fibrillation. Circ Arrhythm Electrophysiol. 2021;14(3):e009368. 33657832 10.1161/CIRCEP.120.009368

[22] Park JW , Yu HT , Kim TH , Uhm JS , Joung B , Lee MH , et al. Atrial fibrillation catheter ablation increases the left atrial pressure. Circ Arrhythm Electrophysiol. 2019;12(4):e007073. 30917688 10.1161/CIRCEP.118.007073

[23] Park J , Joung B , Uhm JS , Young Shim C , Hwang C , Hyoung Lee M , et al. High left atrial pressures are associated with advanced electroanatomical remodeling of left atrium and independent predictors for clinical recurrence of atrial fibrillation after catheter ablation. Heart Rhythm. 2014;11(6):953–960. 24607916 10.1016/j.hrthm.2014.03.009

[24] Yu HT , Shim J , Park J , Kim IS , Kim TH , Uhm JS , et al. Pulmonary vein isolation alone versus additional linear ablation in patients with persistent atrial fibrillation converted to paroxysmal type with antiarrhythmic drug therapy: a multicenter, prospective, randomized study. Circ Arrhythm Electrophysiol. 2017;10(6):e004915. 28611206 10.1161/CIRCEP.116.004915

[25] Calkins H , Kuck KH , Cappato R , Brugada J , Camm AJ , Chen SA , et al. 2012 HRS/EHRA/ECAS expert consensus statement on catheter and surgical ablation of atrial fibrillation: recommendations for patient selection, procedural techniques, patient management and follow‐up, definitions, endpoints, and research trial design: a report of the Heart Rhythm Society (HRS) Task Force on Catheter and Surgical Ablation of Atrial Fibrillation. Developed in partnership with the European Heart Rhythm Association (EHRA), a registered branch of the European Society of Cardiology (ESC) and the European Cardiac Arrhythmia Society (ECAS); and in collaboration with the American College of Cardiology (ACC), American Heart Association (AHA), the Asia Pacific Heart Rhythm Society (APHRS), and the Society of Thoracic Surgeons (STS). Endorsed by the governing bodies of the American College of Cardiology Foundation, the American Heart Association, the European cardiac arrhythmia society, the European heart rhythm association, the Society of Thoracic Surgeons, the Asia Pacific Heart Rhythm Society, and the Heart Rhythm Society. Heart Rhythm. 2012;9(4):632–696.e21. 22386883 10.1016/j.hrthm.2011.12.016

[26] Hughes RK , Knott KD , Malcolmson J , Augusto JB , Mohiddin SA , Kellman P , et al. Apical hypertrophic cardiomyopathy: the variant less known. J Am Heart Assoc. 2020;9(5):e015294. 32106746 10.1161/JAHA.119.015294 PMC7335568

[27] Choi HM , Kim KH , Lee JM , Yoon YE , Lee SP , Park EA , et al. Myocardial fibrosis progression on cardiac magnetic resonance in hypertrophic cardiomyopathy. Heart. 2015;101(11):870–876. 25897040 10.1136/heartjnl-2014-306555

[28] Hoit BD . Left atrial reservoir strain. JACC Cardiovasc Imaging. 2022;15(3):392–394. 34801456 10.1016/j.jcmg.2021.10.003

[29] Penela D , Sorgente A , Cappato R . State‐of‐the‐art treatments for atrial fibrillation in patients with hypertrophic cardiomyopathy. J Clin Med. 2021;10(14):3025. 34300191 10.3390/jcm10143025 PMC8303743

