# Supplementary material for: Clinical and pathophysiologic determinants of catheter ablation outcome in hypertrophic cardiomyopathy with atrial fibrillation
Source: J Arrhythm. 2024 May 14;40(3):479–88. doi: 10.1002/joa3.13061 (PMC11199821; doi:10.1002/joa3.13061)
Supplement: Supplementary file 1 — Appendix S1. [file JOA3-40-479-s001.docx]

**SUPPLEMENTAL MATERIAL**

**Supplementary Figure 1. AF recurrence outcome in AF-HCM group according to the type of AF.**

**Supplementary Figure 2. AF recurrence outcome in (A) persistent AF and (B) paroxysmal AF among three groups.**

**Supplementary Figure 3. AF recurrence outcome in repeat procedure between AF-HCM and control groups.**

**Supplementary Table 1. Regional LA wall thickness between AF-HCM and control group.**

**Supplementary Table 2. Echocardiographic findings and changes in subjects with no AF recurrence.**

**Supplementary Table 3. Echocardiographic findings and changes in subjects with AF recurrence.**

**Supplementary Table 4. Cox regression analysis for AF recurrence in HCM subjects.**

**Supplementary Table 5. Repeat procedural findings in the two groups.**


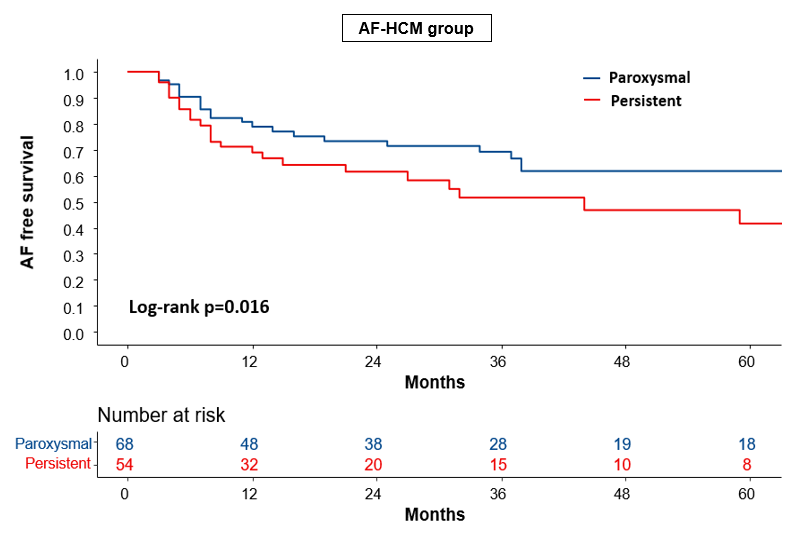


**Supplementary Figure 1. AF recurrence outcome in AF-HCM group according to the type of AF.**


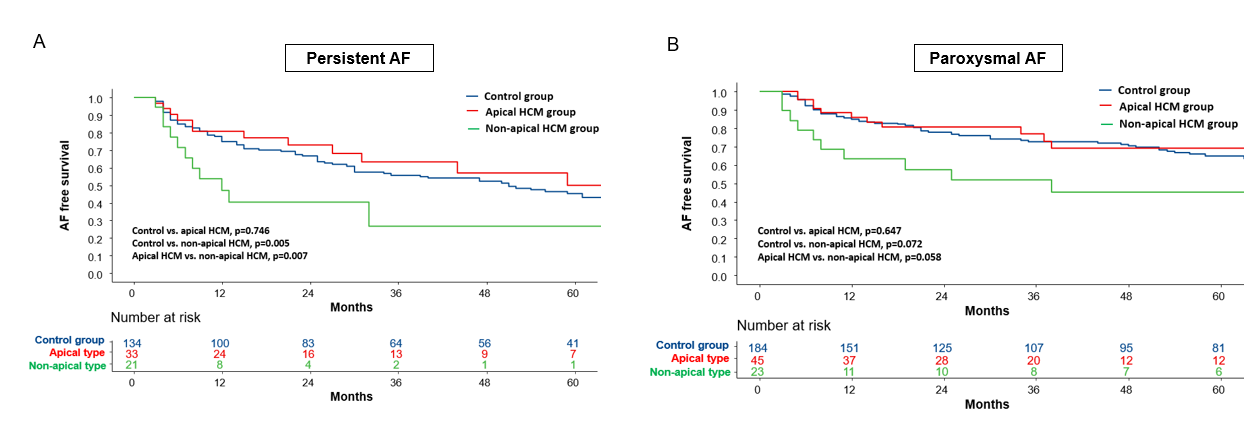


**Supplementary Figure 2. AF recurrence outcome in (A) persistent AF and (B) paroxysmal AF among three groups.**


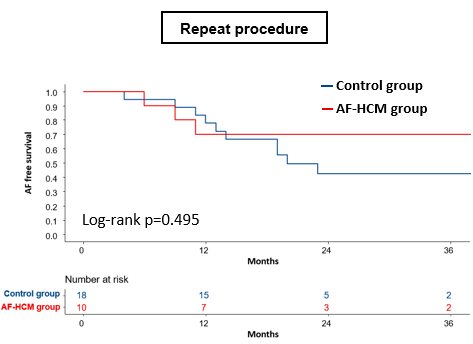


**Supplementary Figure 3. AF recurrence outcome in repeat procedure between AF-HCM and control groups.**

**Supplementary Table 1. Regional LA wall thickness between AF-HCM and control group.**

|  | All subjects  (n=440) | AF-HCM  (n=122) | Control  (n=318) | p |
| --- | --- | --- | --- | --- |
| Regional LA wall thickness | | | | |
| Anterior wall | 1.94 (1.66–2.22) | 1.96 (1.67–2.25) | 1.87 (1.61–2.17) | 0.117 |
| Posterior wall | 1.84 (1.60–2.06) | 1.85 (1.61–2.03) | 1.81 (1.52–2.09) | 0.553 |
| Posterior-inferior wall | 1.60 (1.38–1.85) | 1.63 (1.40–1.85) | 1.53 (1.34–1.83) | 0.097 |
| Interatrial septum | 2.39 (1.99–2.72) | 2.40 (1.99–2.74) | 2.33 (2.00–2.69) | 0.395 |
| Left superior PV | 2.06 (1.77–2.37) | 2.05 (1.78–2.37) | 2.07 (1.71–2.39) | 0.982 |
| Left inferior PV | 2.19 (1.90–2.53) | 2.19 (1.92–2.55) | 2.18 (1.84–2.51) | 0.417 |
| Right superior PV | 1.78 (1.52–2.01) | 1.80 (1.52–2.02) | 1.73 (1.47–1.97) | 0.232 |
| Right inferior PV | 1.73 (1.50–2.03) | 1.74 (1.51–2.08) | 1.72 (1.45–1.97) | 0.099 |
| Left lateral isthmus | 2.29 (2.00–2.65) | 2.32 (2.01–2.63) | 2.21 (1.98–2.69) | 0.841 |
| LA appendage | 2.13 (1.88–2.40) | 2.12 (1.86–2.38) | 2.18 (1.94–2.46) | 0.160 |

Values are presented as median (Q1–Q3 quartiles [25th and 75th percentiles]).

AF-HCM, atrial fibrillation with hypertrophic cardiomyopathy; LA, left atrium; PV, pulmonary vein.

**Supplementary Table 2. Echocardiographic findings and changes in subjects with no AF recurrence.**

|  | Not recurred subjects  (n=235) | Control  (n=162) | AF-HCM  (n=73) | p |
| --- | --- | --- | --- | --- |
| **Baseline echocardiography** | |  |  |  |
| LA dimension (mm) | 42.4 (38.0–46.0) | 41.0 (37.0–45.0) | 46.0 (41.5–50.5) | <0.001 |
| LV ejection fraction (%) | 65.0 (60.0–71.0) | 64.0 (59.0–71.0) | 68.0 (63.5–74.0) | 0.001 |
| E/Em | 11.0 (8.3–14.0) | 10.0 (8.0–12.0) | 14.9 (10.0–18.5) | <0.001 |
| **1-year f/u echocardiography** | |  |  |  |
| LA dimension (mm) | 39.0 (35.0–43.0) | 38.0 (34.5–41.0) | 42.0 (39.0–46.0) | <0.001 |
| LV ejection fraction (%) | 65.0 (61.0–70.0) | 64.0 (60.0–69.5) | 67.0 (62.0–71.0) | 0.121 |
| E/Em | 10.0 (8.0–13.8) | 9.3 (8.0–11.6) | 14.9 (10.4–20.2) | <0.001 |
| **1-year change in the parameters compared to baseline echocardiography** | | | | |
| ΔLA dimension (mm) | -7.6 (-13.3–0.0) | -8.6 (-14.3– 2.3) | -6.4 (-10.2–-2.3) | 0.536 |
| ΔLV ejection fraction (%) | 0.7 (-5.2–9.2) | 1.7 (-4.8–10.1) | 0 (-7.1– 4.5) | 0.240 |
| ΔE/Em | 0 (-15.4–21.7) | 0 (-16.3–20.0) | 11.3 (-14.1–26.2) | 0.121 |

Values are presented as median (Q1–Q3 quartiles [25th and 75th percentiles]).

AF-HCM, atrial fibrillation with hypertrophic cardiomyopathy; E/Em, ratio of the peak mitral flow velocity of the early rapid filling to the early diastolic velocity of the mitral annulus; LA, left atrium; LV, left ventricular.

**Supplementary Table 3. Echocardiographic findings and changes in subjects with AF recurrence.**

|  | Recurred subjects  (n=205) | Control  (n=156) | AF-HCM  (n=49) | p |
| --- | --- | --- | --- | --- |
| **Baseline echocardiography** | |  |  |  |
| LA dimension (mm) | 45.0 (41.0–49.0) | 44.0 (41.0–48.0) | 47.0 (42.0–52.0) | 0.002 |
| LV ejection fraction (%) | 63.0 (59.0–69.0) | 62.5 (58.0–68.5) | 66.0 (62.0–70.0) | 0.064 |
| E/Em | 10.2 (8.0–14.0) | 10.0 (8.0–13.0) | 13.0 (10.4–18.0) | <0.001 |
| **1-year f/u echocardiography** | |  |  |  |
| LA dimension (mm) | 41.0 (38.0–45.0) | 40.0 (37.0–44.0) | 46.0 (42.5–49.5) | <0.001 |
| LV ejection fraction (%) | 65.0 (61.0–69.0) | 64.0 (60.0–69.5) | 66.0 (61.5–69.0) | 0.401 |
| E/Em | 10.8 (8.2–16.0) | 10.0 (8.0–14.1) | 14.0 (12.1–21.1) | <0.001 |
| **1-year change in the parameters compared to baseline echocardiography** | | | | |
| ΔLA dimension (mm) | -6.0 (-13.2–0.0) | -7.3 (-14.3– 0.0) | 0 (-5.9– 2.3) | <0.001 |
| ΔLV ejection fraction (%) | 1.6 (-5.6–10.3) | 2.5 (-5.6–10.5) | 0 (-3.2– 6.6) | 0.544 |
| ΔE/Em | 4.2 (-12.8–29.4) | 0 (-13.0–29.0) | 8.3 (-5.0–36.1) | 0.342 |

Values are presented as median (Q1–Q3 quartiles [25th and 75th percentiles]).

AF-HCM, atrial fibrillation with hypertrophic cardiomyopathy; E/Em, ratio of the peak mitral flow velocity of the early rapid filling to the early diastolic velocity of the mitral annulus; LA, left atrium; LV, left ventricular.

**Supplementary Table 4. Cox regression analysis for AF recurrence in HCM subjects.**

|  | Multivariate | |
| --- | --- | --- |
|  | Adjusted HR (95% CI) ^a^ | p |
| Non-apical HCM type | 2.28 (1.26–4.16) | 0.007 |
| Persistent AF | 2.02 (1.09–3.74) | 0.025 |

^a^HRs were adjusted for non-apical type HCM, persistent AF, and LA reservoir strain those were with p-value <0.05 in the univariate analysis.

AF, atrial fibrillation; CI, confidence interval; HCM, hypertrophic cardiomyopathy; HR, hazard ratio.

**Supplementary Table 5. Repeat procedural findings in the two groups.**

|  | Repeat procedure  (n=28) | AF-HCM  (n=10) | Control  (n=18) | p |
| --- | --- | --- | --- | --- |
| Age (year) | 65.5 (60.5–70.0) | 63.0 (56.0–70.0) | 66.0 (63.0–70.0) | 0.336 |
| Male sex, n (%) | 24 (85.7) | 9 (90.0) | 15 (83.3) | >0.999 |
| Paroxysmal AF, n (%) | 13 (46.4) | 3 (30.0) | 10 (55.6) | 0.366 |
| Redo-procedure interval (months) | 56.0 (21.0–86.0) | 42.0 (23.0–72.0) | 66.0 (19.0–101.0) | 0.517 |
| **Redo-ablation lesion, n (%)** | | | | |
| CPVI | 28 (100) | 10 (100) | 18 (100) | 1 |
| Posterior box isolation | 22 (78.6) | 9 (90.0) | 13 (72.2) | 0.537 |
| Left lateral isthmus line | 3 (10.7) | 0 (0) | 3 (16.7) | 0.466 |
| Anterior line | 16 (57.1) | 6 (60.0) | 10 (55.6) | >0.999 |
| Extra PV foci, n (%) | 9 (32.1) | 2 (20.0) | 7 (38.9) | 0.546 |
| PV reconnection, n (%) | 20 (71.4) | 8 (80.0) | 12 (66.7) | 0.755 |
| 1–2 PV reconnection | 14 (50.0) | 6 (60.0) | 8 (44.4) | 0.693 |
| 3–4 PV reconnection | 6 (21.4) | 2 (20.0) | 4 (22.2) | >0.999 |
| Early recurrence, n (%) | 8 (28.6) | 3 (30.0) | 5 (27.8) | >0.999 |
| Clinical recurrence, n (%) | 13 (46.4) | 3 (30.0) | 10 (55.6) | 0.366 |
| Mean LA voltage at redo-procedure (mV) | 1.3 (0.7–2.0) | 2.1 (0.6–2.4) | 1.1 (0.8–1.9) | 0.506 |
| LA peak pressure at redo-procedure (mmHg) | 29.0 (23.0–43.0) | 32.0 (28.5–41.5) | 29.0 (22.0–37.0) | 0.230 |

Values are presented as median (Q1–Q3 quartiles [25th and 75th percentiles]) or number (%).

AF, atrial fibrillation; AF-HCM, atrial fibrillation with hypertrophic cardiomyopathy; CPVI, circumferential pulmonary vein isolation; LA, left atrium; PV, pulmonary vein.
